# 808. Autologous Platelet-rich Plasma versus Modified Medium for in vitro Keratinocyte Growth

**DOI:** 10.1093/jbcr/irag033.186

**Published:** 2026-04-06

**Authors:** Wayne George Kleintjes, Tarryn Kay Prinsloo

**Affiliations:** Sheik Khalifa Hospital Fujairah, Cape Town, Western Cape; Cape Peninsula University of Technology, Cape Town, Western Cape

## Abstract

**Introduction:**

Cultured epidermal autografts (CEA) require daily supplementation with autologous platelet-rich plasma (PRP) during the two-week culture period to promote keratinocyte growth and prevent desiccation. However, repeated blood withdrawal may increase morbidity, particularly in vulnerable patient populations such as pediatric or critically ill burn patients. Modified mediums are widely available in laboratories and could provide a feasible alternative or adjunct. We hypothesized that keratinocytes cultured with a modified medium would demonstrate growth patterns comparable to those supported by PRP, potentially reducing reliance on daily blood-derived supplementation.

**Methods:**

An observational comparative in vitro pilot study was designed to compare the growth of keratinocytes, typically prepared for CEA cultivation with a modified technique, in response to dilutions of modified medium and PRP. Keratinocytes were enzymatically retrieved from full-thickness skin biopsies using trypsin. They were then immersed in serially diluted suspensions of PRP and modified medium (0–100%, v/v) prior to cultivation, and incubated in pediatric incubators at 37°C for seven days. Cell growth was compared following macroscopic evaluation of suspension volume, viscosity and density (cell-to-gel ratios).

**Results:**

Keratinocytes cultured with PRP demonstrated superior gel formation and higher cell-to-gel ratios, with growth appearing visible, opaque, and dense, compared with modified medium, where growth was less visible, translucent, and fluidic. The highest cell yields in PRP occurred at the lowest dilution, although growth remained evident at concentrations containing as little as 50% PRP.

**Conclusions:**

These preliminary findings suggest that PRP remains superior to the modified medium in supporting keratinocyte proliferation and organization during early culture. This may be attributed to the presence of PRP-derived cytokines and growth factors such as platelet derived-, vascular endothelial-, and epidermal growth factors. While the modified medium supported some cell survival, cultures lacked the density and structural maturation seen with PRP supplementation. Therefore, the partial growth observed with the modified medium suggests that optimizing medium-based or combined supplementation strategies may reduce dependence on daily PRP preparation.

**Applicability of Research to Practice:**

If refined, modified medium-based supplementation could reduce or eliminate the need for repeated blood withdrawal during CEA culture, thereby lowering morbidity in vulnerable patients and streamlining laboratory workflows. Such strategies may improve the feasibility of CEA cultivation in both high- and low-resource settings, expanding access to this technique.

**Funding for the study:**

N/A.

